# Mesoscopic Model and Free Energy Landscape for Protein-DNA Binding Sites: Analysis of Cyanobacterial Promoters

**DOI:** 10.1371/journal.pcbi.1003835

**Published:** 2014-10-02

**Authors:** Rafael Tapia-Rojo, Juan José Mazo, José Ángel Hernández, María Luisa Peleato, María F. Fillat, Fernando Falo

**Affiliations:** 1 Dpto. de Física de la Materia Condensada, Universidad de Zaragoza, Zaragoza, Spain; 2 Institute for Biocomputation and Physics of Complex Systems, Zaragoza, Spain; 3 Instituto de Ciencia de Materiales de Aragón, C.S.I.C.-Universidad de Zaragoza, Zaragoza, Spain; 4 Department of Biochemistry, Midwestern University, Glendale, Arizona, United States of America; 5 Departamento de Bioquímica y Biología Molecular y Celular, Universidad de Zaragoza, Zaragoza, Spain; Stanford University, United States of America

## Abstract

The identification of protein binding sites in promoter sequences is a key problem to understand and control regulation in biochemistry and biotechnological processes. We use a computational method to analyze promoters from a given genome. Our approach is based on a physical model at the mesoscopic level of protein-DNA interaction based on the influence of DNA local conformation on the dynamics of a general particle along the chain. Following the proposed model, the joined dynamics of the protein particle and the DNA portion of interest, only characterized by its base pair sequence, is simulated. The simulation output is analyzed by generating and analyzing the Free Energy Landscape of the system. In order to prove the capacity of prediction of our computational method we have analyzed nine promoters of *Anabaena* PCC 7120. We are able to identify the transcription starting site of each of the promoters as the most populated macrostate in the dynamics. The developed procedure allows also to characterize promoter macrostates in terms of thermo-statistical magnitudes (free energy and entropy), with valuable biological implications. Our results agree with independent previous experimental results. Thus, our methods appear as a powerful complementary tool for identifying protein binding sites in promoter sequences.

## Author Summary

Binding of specific proteins to particular sites in the DNA sequence is a fundamental issue for gene regulation in molecular biology and genetic engineering. A deep understanding of cell physiology requires the analysis of a plethora of genes involving characterization of their promoter architectures that determine their regulation and gene transcription. In order to locate the promoter elements of a given gene, experimental determination of its transcription start site (TSS) is required. This is an expensive, time-consuming task that, depending on our requirements, could be simplified using computational analysis as a first approach. Nevertheless, most computational methods lack a physical basis on the protein-DNA interaction mechanism. We adopt here this strategy, by using a simple model for protein-DNA interaction to find TSS in a bunch of cyanobacteria promoters. We make use of physical tools to characterize these TSS and to relate them with biological properties as the relative strength of the promoter. Our study shows how a model based on a coarse-grained description of a biomolecule can give valuable insight on its biological function.

## Introduction

Transcriptional regulation is the main mechanism for gene control in prokaryotes. In order to adapt optimal protein expression to nutritional and environmental conditions, a cascade of transcriptional regulators works as signal transducers determining the accessibility of RNA polymerase to bacterial promoters. In the last years, high throughput approaches have been confirmed as powerful tools for a better understanding of the regulatory networks that govern key aspects of cell physiology, such as the mechanisms leading to pathogenesis or the acclimation to xenobiotics and hostile environments, among others [1]–[4].

However, successful transcriptome sequencing requires the generation of comprehensive transcriptome profiles that rely on the isolation of a sufficiently large number of reads to detect those biologically relevant transcripts, that represent a relatively small proportion of the cDNA library [5]. Moreover, those procedures are time consuming and, in many cases, the budget for sequencing costs constrains the total number of reads that can be obtained [6], [7].

Therefore, computational methods emerge as valuable complementary approaches for prediction or further validation of high throughput results [8], [9]. Mostly, a statistical approach to the study of sequences is adopted, leading to a general lack of methods based on the physical mechanism of protein-DNA interactions. A possibility to tackle the problem is the microscopic study of protein-DNA interaction [10]–[12], but this approach demands huge computer facilities and it is restricted to few base pairs up to the date. In this sense, coarse-grained models arise as powerful tools to model biological systems, speeding up the computation and allowing to get a deeper insight in the physical interactions [13], [14]. Adopting this strategy, we develop a coarse-grained model that allows for the analysis of promoter sequences and the identification and characterization of protein binding sites, likely related to transcriptional activity in the genome of the nitrogen-fixing cyanobacterium *Anabaena* PCC 7120.

Cyanobacteria are the only prokaryotes able to perform oxygenic photosynthesis, being key contributors to 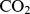 fixation. The ability of some cyanobacterial strains to fix atmospheric nitrogen or the formation of harmful blooms by toxigenic species, among other properties, evidence their ecological relevance [15]. Besides, cyanobacteria are an excellent model for the study of multicellularity in prokaryotes [16] and potential sources for novel drugs derived from their secondary metabolites [17].

The genome of *Anabaena* PCC 7120 contains 7,211,789 base pairs (bp) and 6,223 genes organized in a 6,413,771 bp chromosome and 6 plasmids [18]. *Anabaena* PCC 7120 has been used for long time as a model for the study of prokaryotic cell differentiation and nitrogen fixation [19]. More recently, the experimental definition of a genome wide map of transcriptional start sites (TSSs) of *Anabaena* together with the analysis of transcriptome variations resulting from the adaptation to nitrogen stress have provided a holistic picture of this complex process [20].

The problem of protein-DNA recognition is a widely debated issue, yet far to be fully understood. In this sense, it has been widely reported how the physical properties of the DNA chain result in key functional consequences in this process. DNA local structure highly influences some transcription factors (TFs) binding [21]–[23]. Thermal stability and bubble formation (*i.e.* local long-lived transient openings in the DNA strands) has also been extensively reported to correlate with several DNA functions, such as the recombination rate, single nucleotide polymorphism, DNA replication or gene transcription [24]–[27]. In this regard, the relation between bubble formation and the location of protein binding sites, is a lengthly, controversial debate, greatly nourished by the study of Peyrard-Bishop-Daxouis (PBD) model [28], [29]. This mesoscopic model was initially intended to reproduce the DNA melting transition, though it has been widely used afterwards for studying bubble formation on DNA promoters, likely correlated with biological relevant sites in the sequence, such as the TSS or the TATA box [30]–[35].

Despite the lack of consensus on whether PBD model is suitable for predicting protein binding sites [36]–[39], strong evidence supports this idea, showing clear correlation between regions with high propensity to form bubbles, and the presence of binding sites of DNA-interacting proteins such as RNA polymerase, [30]–[32], [40] or some TFs [33], [34], [41], [42]. Even more, succeeding revisions of this model showed clear relation between flexibility profiles and location of TSSs [43]. Grounded on these evidences, we propose a physical model for protein-DNA interaction in promoters [44], based on the coupling of a generic particle with the sequence-dependent bubble formation. This simple model is combined with a suitable analysis method [45] allowing the detection of biologically relevant sites, namely TSSs, on promoters of a prokaryote genome.

In order to prove the capacity of prediction of the computational methods developed in [44] and [45] for identifying the TSSs of a promoter, we have analyzed the result of simulating the dynamics of nine promoters of *Anabaena* PCC 7120. We have analyzed the simulations outputs and built systematically the relevant macrostates of the system. In every case, our analysis algorithm finds the TSS as one of these states, yielding in addition thermodynamic parameters (*e.g.* free energy, entropy) that allow their physical characterization and thus further biological discussion. In this regard, our method arises as a complementary tool that, from physical principles, finds protein binding sites (we focus on TSSs) and characterizes them, allowing to discuss the strength -in terms of RNA production- of such sites, something not achievable by statistical methods. Remarkably, in this case the base pair sequence is the only previous information required. Thus, our numerical outcomes are independent numerical predictions to be confronted with previous or future experimental results.

## Methods

### Model

**Figure 1:**
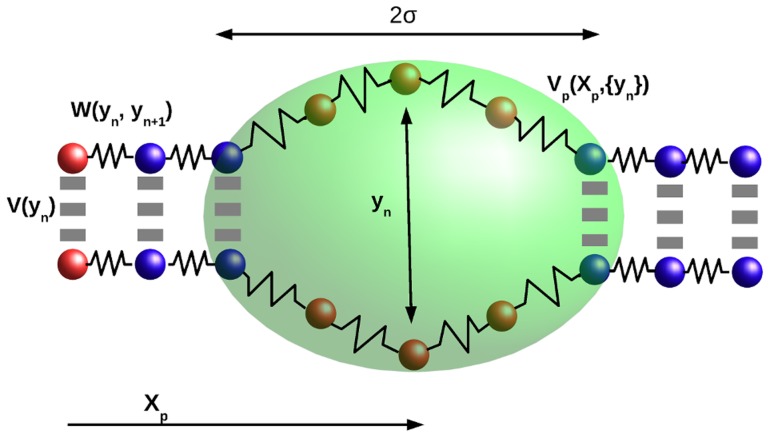
Simplified illustration of the DNA-particle interaction model. The one-dimensional chain (solid spheres) models the DNA chain considering a single relevant degree of freedom 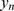 per base pair, and two phenomenological potentials [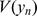 and 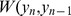]. The brownian particle, with coordinate 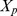 (dim ellipse), diffuses along the chain interacting with open regions through the potential 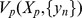.

We base our model on a modification of the PBD model [28]–[31], [35] to include the interaction with a generic particle as a sliding protein coupled with the sequence. PBD model reduces the complexity of DNA to a set of 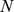 units that represent the 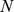 base pairs of the chain (see Fig. 1). The only degrees of freedom are the coordinates 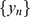 which stand for the opening of each base pair. The total Hamiltonian of the model accounts for two phenomenological interactions, the intra-base 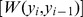 and the inter-base 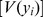 potentials, 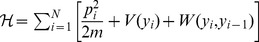, where 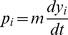 is the linear momentum of the 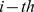 base pair and 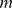 its reduced mass.

The potential 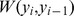 describes the inter-base pair or *stacking* interactions. The election is the anharmonic potential [28] 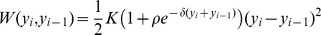 whose elastic constant is 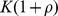 for small openings but drops to 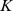 for large 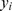. The parameter 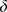 sets the length scale for this behavior.

The original PBD model uses Morse potential for the intra-base pair interaction. Nevertheless, a successful modification includes an entropic barrier which accounts for solvent interactions with open base pairs [35], [46], [47]. This modification sharpens the thermal denaturation and stabilizes the bubbles, reproducing in a more realistic way the experiments [35], [46], [47]. We include this effect adding a gaussian barrier [35], thus 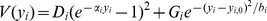. Sequence dependence is introduced only in this potential term as the interaction is stronger if the base pair is C-G than if it is A-T (see Text S1 for the complete set of parameters). Sequence-dependence can be also introduced in the stacking potential parameters, a modification that accounts for flexibility properties of the DNA chain [40], [43], [48].

Inspired on the one-dimensional diffusion stage of DNA-interacting proteins [49], we include a new degree of freedom to the traditional PBD model. This new degree of freedom consists on a brownian particle that moves along the DNA chain (see Fig. 1 for a schematic representation of the total system) interacting with it through a phenomenological potential which depends on 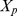, the coordinate of the Brownian particle along the DNA molecule, and the DNA instantaneous configuration 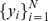


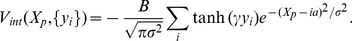
(1)


This potential creates a classical field composed by a sum of gaussian wells centered at each base (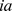) and whose amplitude depends on the opening of the base pair. The 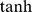 term allows a linear dependence for low 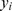 saturating the interaction for large 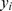 in order to avoid self-trapping. In this sense, the particle interacts more intensely with open regions of the sequence. In addition, the base pairs are also affected by the particle, so that they will be more likely to be opened if the particle is within its range of interaction. The model introduces only three new parameters, as the longitudinal scale over which the particle slides is adimensional (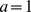). The interaction intensity 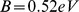 and width 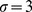 are set so that bubbles span around 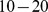 base pairs, an adequate value for the kind of processes studied here [50]. The parameter 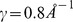 saturates the interaction around 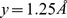, typical value for open base-pairs [50]–[52].

### Langevin dynamics simulations

The model is simulated by integrating numerically the Langevin equations for the chain base pairs and the particle using the stochastic Runge-Kutta algorithm of fourth order [53] (see Text S1 for explicit formulation of the equations of motion). Each of the DNA sequences we study is simulated in five different realizations, each one covering 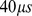, with a preheating time of 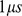. For sequences up to 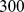 base pairs, these times are enough to ensure equilibrium and ergodicity. In addition, since one-dimensional diffusion times of binding proteins are in the range of milliseconds, our simulation times are reasonable from a biological perspective. The simulation temperature is 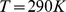. We use periodic boundary conditions for the diffusing particle and fixed boundary conditions for the sequence, adding 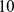 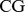 base pair clamps at the end of each sequence to provide “hard-boundaries” and avoid undesirable end effects. Relevant observables from the trajectories can be obtained, mainly the base pairs mean position 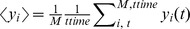, where 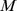 is the number of realizations and 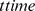 the simulation time of each realization, and the particle's trajectory histogram.

### Principal Component Analysis (PCA)

The large dimensionality of the system requires a method to reduce the number of coordinates while keeping the relevant information of study. PCA [54] is one the most popular methods to reduce systematically the dimensionality of a complex system. PCA performs a linear transformation by diagonalizing the covariance matrix 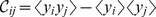, and thus removing all internal correlations. It has been proved that, by ordering the eigenvalues decreasingly, the few first principal components contain most of the fluctuations of the system, and thus can be chosen as convenient reaction coordinates [35], [55], [56].

We project the 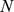 base pair trajectories into the first five eigenspaces, describing thus the system in terms of the first five principal components and the particle trajectory. With this choice we keep over the 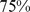 of the fluctuations.

### Conformational Markov Network

The Conformational Markov Network has been proven to be a useful and powerful tool to analyze trajectories from high dimensional systems, such as those from Molecular Dynamics simulations [45], [57]–[59]. This representation is obtained by discretizing the conformational space explored by the system in order to build a complex network. Each node in the network represents a discretized region of the conformational space, a conformational microstate, weighted according to the fraction of trajectory visiting such microstate. The links of the network coincide with the observed transitions between microstates, and are thus directed and weighted. We build the Conformational Markov Network of our system by considering the 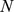 posible positions of the particle along the chain, and binning each of the five principal components into 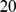 bins.

### Finding macrostates

Typically, the Conformational Markov Network is formed by a large number of nodes which prevent a direct interpretation of the results. In order to extract relevant information about the physical states of the system and its relevance in the dynamics, we split the network into its basins of attraction, i.e. regions in which the probability fluxes (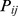) converge to a common state (attractor) of the network. To do so, we apply the stochastic steepest descent algorithm, developed in [45], building a coarse grained representation of the former network. From this basin network, the Free Energy Landscape (FEL) can be represented as a hierarchical tree diagram (dendrogram or disconnectivity graph) [60], [61], by assigning to each node a free energy according to its weight 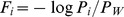 where 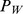 is the weight of the heaviest basin. This magnitude is used as a control parameter, increasing it step by step from the weightiest node, so that new nodes arise, together with their links (see Text S1 for a more explicit exposition of the algorithm). The disconnectivity graph represents each basin of attraction hierarchically ordered according to its free energy, while the connections among them stand for the barriers needed to jump from to another (see below and Text S1 for plots of the disconnectivity graphs or dendrograms).

We define now the macrostates 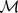 of the system by clustering every basin separated by a free energy barrier lower than 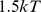, as the system transits among them within short waiting times. In fact, we can check how they represent qualitatively similar physical configurations. Each macrostate 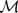 has an assigned weight 
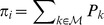
. We want to calculate free energy differences between specific and non-specific states. The basin network contains a huge number of low populated states, see [35], that constitute transitionary states between well defined attractors of the system. Physically, they are short-lived transitionary states where the particle diffuses until it binds to a target site. We determine these non-specific states as every basin with a population 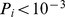 and calculate free energy differences between specific and non-specific states as 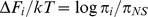, where 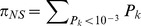 is the total weight of all non-specific states. In addition, we define the entropy of a macrostate 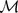 as 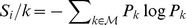.

## Results

We have analyzed nine promoter sequences from *Anabaena* PCC 7120 which exhibit different features. Using our computational approach, we have to identify the TSSs in the promoter sequences as sites where bubbles form with high probability. Within the frame of our model, this is reflected in larger openings of the chain at these sites and higher probability of the particle to visit them. Next, we apply the analysis algorithm to define the macrostates of the system and extract the FEL as a dendrogram or disconnectivity graph [59], [60]. This procedure allows us to characterize these states in order to extract solid conclusions about each sequence. The strength of each TSS can be determined and, if the sequence presents more than one TSS, their relative strength can be compared, obtaining useful biological conclusions.

### PCA analysis of complete genes

Up to our knowledge, most works concerning PBD model limit themselves to the study of short promoter sequences, without justifying the study of this region alone, or how would the model behave in coding regions. In order to cover this gap, we have simulated the behavior of three complete genes from *Anabaena* PCC 7120. We use here the PBD model without including the interacting particle, as we wish just to check in which regions from a whole gene bubbles form more easily. The results allow us to compare the occurrence and intensities of the fluctuations detected in the promoter and the coding regions, validating our further analyses restricted to the promoter sequences.

**Figure 2:**
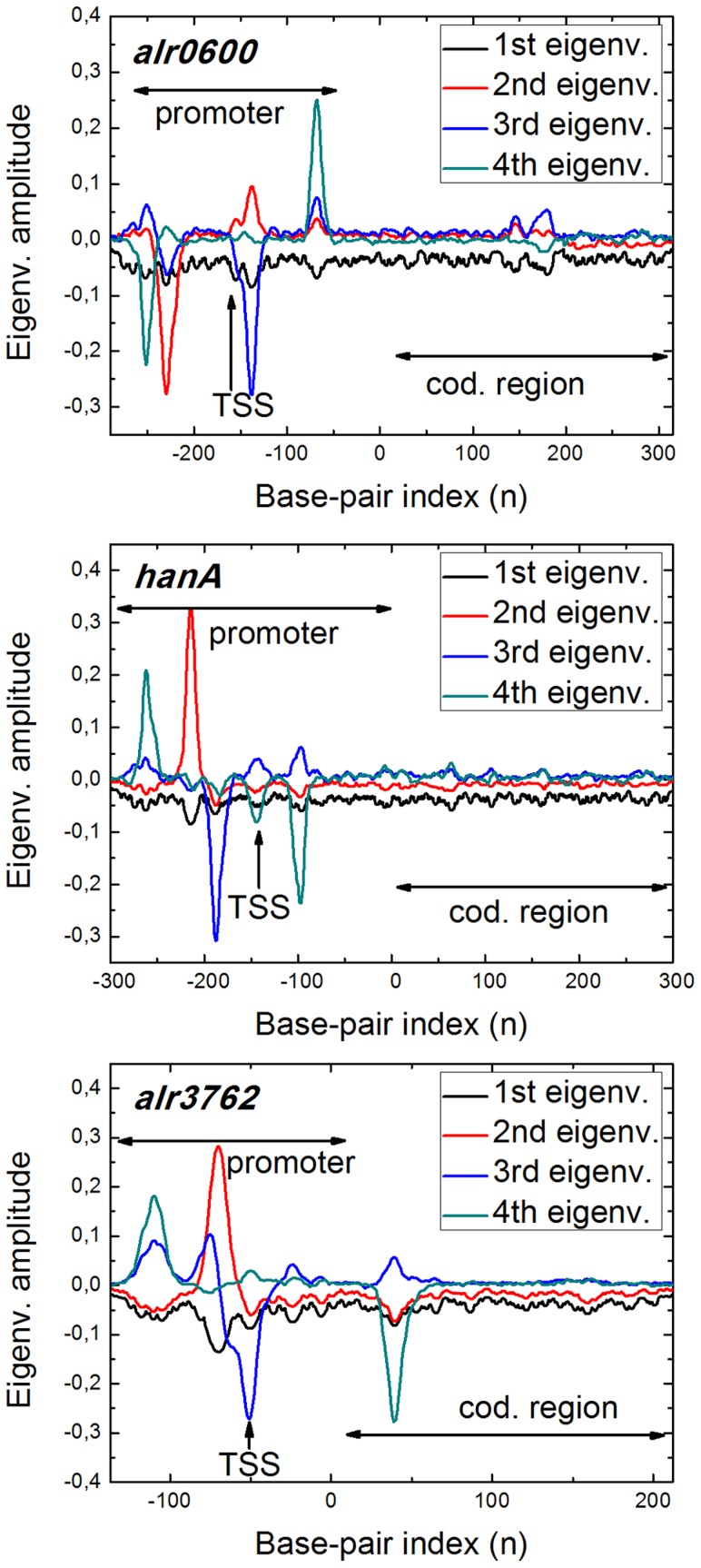
First four PCA eigenvectors calculated for three different complete genes. The promoter region -with the TSS highlighted- and the codifying region are pointed out. Most of the fluctuations appear localized in the promoter region, meaning that bubbles tend to form mostly here. This feature manifests the different mechanical behavior of the promoter and codifying regions, suggesting its key role in the DNA-protein interaction.

Figure 2 shows the first four PCA eigenvectors for the analyzed genes with the promoter and codifying regions highlighted. Very localized eigenvectors indicate strong fluctuations in the region of maximal amplitude. As we can see in Fig. 2, the first eigenvector is delocalized, with small amplitude, accounting for the overall fluctuations of the whole sequence. Nevertheless, the three next eigenvectors are highly localized in specific spots of the sequence. Remarkably, these sites appear in the promoter sequence. Thus, when considering a complete gene within PBD model, most of the system fluctuations occur in the promoter sequence; this is, bubbles form with higher probability there, while the codifying region remains on average closed. This reveals the role of the DNA sequence in the DNA dynamics, and its influence on the DNA-protein interaction problems, supporting strongly that some binding sites in the promoter sequence can be characterized as regions where bubbles form easily, enhancing protein interaction.

### TSS finding and base pair opening

**Figure 3:**
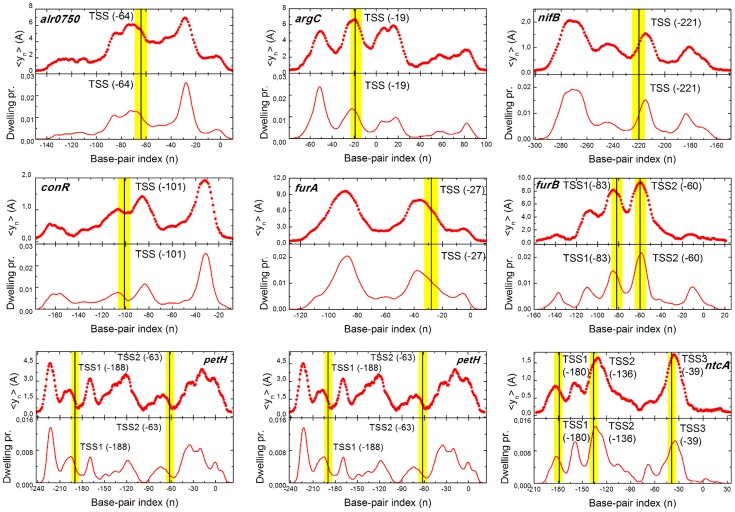
DNA opening versus protein position. Base pair mean opening (upper panels) and particle histogram (lower panels) calculated for each of the nine studied promoters. The horizontal axis represent the base pair positions counted from the coding starting point ATG (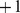). We use this criterion to label the binding sites of the simulated promoters. The experimentally identified TSSs are shaded and their exact location marked with solid bars. In every case a peak appears close to each TSS, meaning this region is “softer” and thus likely to form bubbles, supporting their key role in regulatory processes. The total A-T content of *Anabaena* PCC7120 genome is around 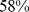 [18]. The A-T content of each analyzed sequence is: *alr0750* (61%); *argC* (64%); *nifB* (68%); *conR* (57%); *furA* (66%); *furB* (65%); *petH* (62%); *petF* (63%); *ntcA* (65%).

We have used the complete model (chain and particle) to analyze nine promoter sequences comprising 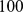 to 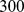 base pairs. In addition, we have chosen promoters with different features, five with a single well characterized TSS (*alr0750*, *argC*, *conR*, *furA* and *nifB*), while four of them exhibit multiple TSSs (*furB*, *ntcA*, *petF* and *petH*) [62]–[69]. Figure 3 shows the base pair opening profile for each promoter sequence with the TSSs highlighted. The particle trajectory histograms are also plotted. In any case, a peak appears close to the TSS, meaning that, on average, bubbles form with high probability around it. In turn, the particle is attracted by this site, as it dwells with high probability around the TSS.

As it has been pointed out in several studies, the PBD model by itself has been successfully used to analyze promoter sequences, finding protein binding sites where bubbles form with high probability, so allowing the identification of TSSs or the TATA-box [30], [32]. Nonetheless, introducing this additional degree of freedom appears as a key feature for our purposes. We are mimicking an hypothetical searching mechanism that indeed affects the dynamics of the system. In the PBD model alone, opening events appear as rare excitations of the unique ground state, where the whole chain is closed. The particle enhances chain opening, stabilizing the bubbles, that last for longer times (around two orders of magnitude longer), enriching the free energy landscape. In addition, bubbles span over a larger number of base pairs, typically around 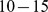, which is a consistent number if we attend to those that form in transcriptional processes [51], [52].

It is also remarkable that the opening probability is not strictly related with the A-T content of the local sequence. Although it is clear that long A-T stretches form “softer” regions in the sequence that can open easier, this intuitive argument does not necessarily applies always. The interplay between the sequence and the dynamics is much more complex. The nonlinearity in the Hamiltonian, the long-range cooperativity of the model and the disorder of the sequence revealed in its heterogeneity affects directly the equilibrium and dynamical behavior of the model, being essential to understand the actual breathing dynamics of DNA, as it has been pointed out in previous studies [30], [31], [40].

Interestingly, besides the peaks centered on the TSSs, other regions exhibit high probability to form bubbles. Many of these peaks correspond to typical regulation sites of bacteria, such as those located at 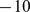 or 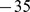 from the TSS, also claimed to be related with bubble formation [30], [40]. These regions appear thus as candidates for possible binding sites of other TFs that are known to be influenced by the physical properties of the DNA chain. Nonetheless, we focus our discussion just on the TSS, as they have been systematically identified in the genome of *Anabaena* PCC 1720.

### FEL analysis

In order to analyze the sequences in a more systematic way we apply the FEL analysis described in the methods section. This algorithm allows us to define the most relevant states in the dynamics characterizing them from a quantitative point of view. So far, we have shown which regions in the promoter sequences exhibit a higher probability to form bubbles and to be visited by the particle. Nonetheless, these magnitudes give just qualitative information, as the average do not inform about the importance of opening events in the system. The real interest of our model and method is the possibility of giving quantitative measures about the “strength” of the different sites in the sequences, specially interesting in those promoters with several TSSs. Each site can be characterized by the thermodynamical magnitudes calculated from the FEL landscape analysis.

We present together the data extracted from the simulation and analysis methods in Table 1. For each of the nine analyzed sequences we show the weight, free energy difference with respect to the non-specific states and the entropy of the TSSs state, all previously defined. We include also other non-identified states in case they appear relevant in the dynamics. Most populated states suppose most stable states, giving rise to high free energies differences. The entropy is the multiplicity of such macro states. Even if the free energy is high, a low entropy would indicate that this macro state is made up of few, yet very populated, basins, physically meaning that the state is very localized (narrow bubbles). The opposite case would indicate that the algorithm finds many, less populated basins that represent the same macrostate. This duality could indicate different regulation behaviors that are further addressed in the Discussion section.

**Table 1:** Thermo-statistical properties of studied promoters.

Sequence	State	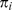	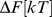	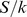
*alr0705*	TSS (−64)	0.219	1.42	0.77
	+28	0.288	1.66	0.85
	NS	0.054	–	–
*argC*	TSS (−19)	0.220	2.10	0.70
	+50	0.329	2.50	0.59
	NS	0.027	-	-
*nifB*	TSS (−221)	0.315	3.47	0.39
	−270	0.444	3.81	0.86
	NS	0.010	-	-
*conR*	TSS (−101)	0.151	1.97	0.58
	−30	0.349	2.80	0.91
	NS	0.021	-	-
*furA*	TSS (−27)	0.449	3.45	1.35
	−87	0.390	3.32	1.16
	NS	0.014	-	-
*furB*	TSS1 (−83)	0.302	2.39	0.86
	TSS2 (−60)	0.276	2.30	0.79
	−10	0.149	1.68	0.28
	NS	0.028	-	-
*petH*	TSS1 (−188)	0.199	3.01	0.74
	TSS2 (−63)	0.117	2.48	0.33
	−220	0.166	2.83	0.40
	NS	0.010	-	-
*petF*	TSS1 (−93)	0.198	3.03	0.58
	TSS2 (−31)	0.268	3.33	0.67
	+1	0.101	2.35	0.33
	NS	0.010	-	-
*ntcA*	TSS1 (−180)	0.098	0.96	0.029
	TSS2 (−136)	0.205	1.69	0.73
	TSS3 (−39)	0.292	2.05	0.85
	NS	0.038	-	-

Occupancy probabilities and thermo-statistical magnitudes of the TSS and other relevant sites of the promoter sequences. NS stands for nonspecific sites defined in the discussion section. As already stated, each site is labelled starting from the 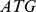 position on the gene (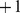).

**Figure 4:**
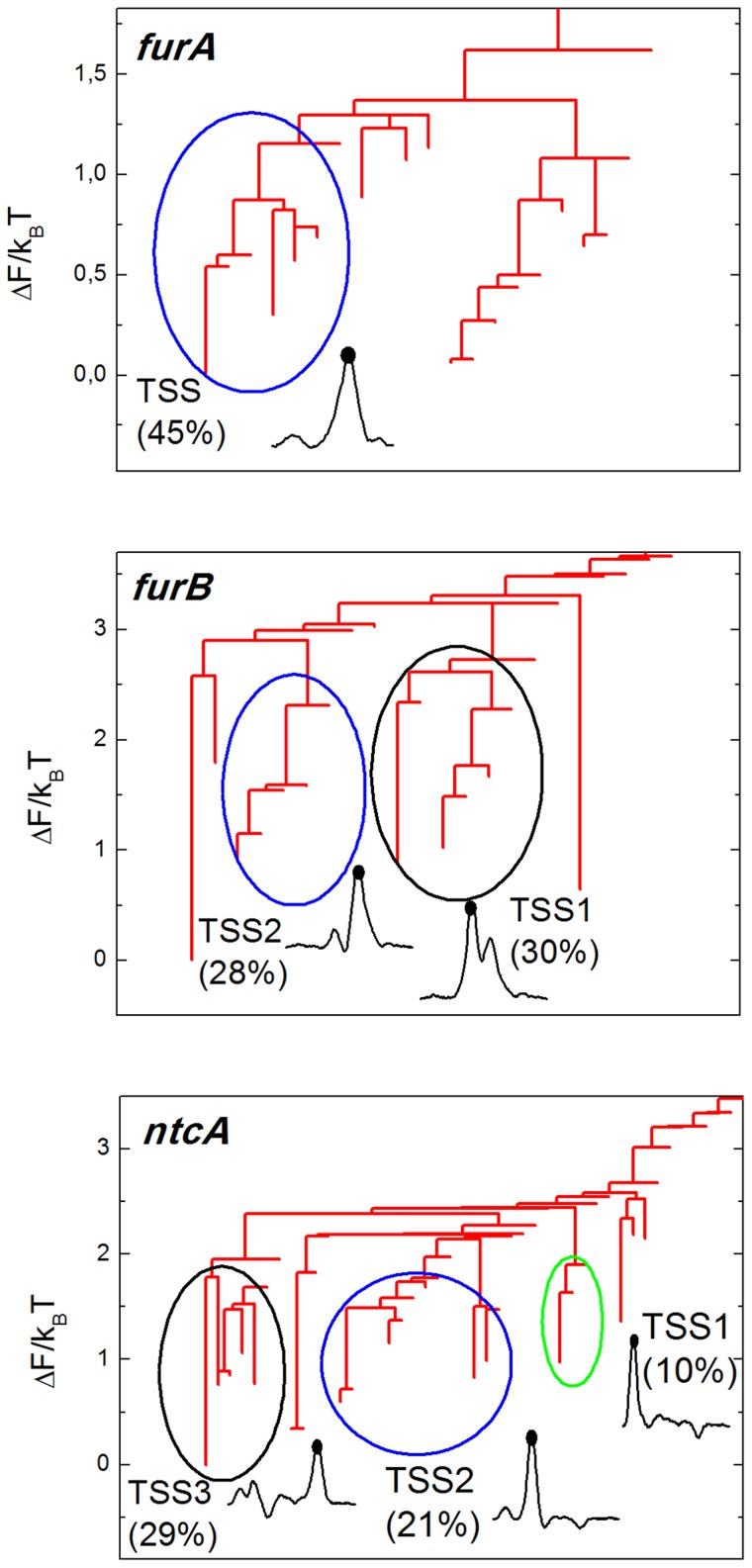
Hierarchical free energy dendrogram for three selected promoters. Basins of attraction separated by barriers lower than 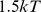 are clustered to define macrostates of the system. Their weight is indicated in the plot together with a representation of the physical state they represent, typically the particle located in a certain site where a bubble opens.

To illustrate the FEL, Fig. 4 shows the free energy dendrograms of three chosen promoters (see Text S1 for the six remaining dendrograms). For the sake of clarity, we do not show the region corresponding to non-specific basins (where 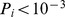, defined above). The position of each basin on the vertical axis informs about its stability, while their hierarchical arrangement about the barrier needed to jump between each state. The dendrogram or disconnectivity graphs provides thus valuable and intuitive information about the thermodynamic and kinetic properties of the FEL of each promoter.

Groups of basins separated by barriers lower than 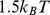 are highlighted by a color circle, defining the macrostates of the system according to the criterion detailed in Methods section. We plot together the physical state associated with it, showing also the fraction of trajectory they occupy. Such states correspond to a large bubble located on the target site, with the particle centered there.

In most cases, the most populated macrostate, and thus the most stable one, coincides with an excitation in the TSS region. Other non identified sites also suppose very populated macrostates, suggesting the possibility of additional regulation sites as it is discussed in next section. Our method arises thus as a powerful tool to complement experimental results, providing additional physical information about the relative importance of these sites in regulation processes.

## Discussion

In this work, we propose the use of a coarse-grained model for protein-DNA interaction to analyze promoter sequences, allowing the detection and characterization of protein-binding sites (we focus on the TSS). The proposed model is based on physical principles and inspired on a relatively simple idea: certain DNA-interacting proteins (as RNA polymerase) couple their binding to DNA bubble dynamics. Due to this, we base our model on a PDB representation of the DNA chain -having been proven to reproduce DNA bubble dynamics successfully- and couple it to an additional degree of freedom representing the protein. In the framework of this model and by using a free energy landscape analysis, we have studied promoters of *Anabaena* PCC7120, allowing the detection and characterization of the TSSs.

Upon genome analysis and TSSs detection, high-throughput approaches, such as proteomics, are commonly used, resulting in an enormous amount of data in a relatively short period of time. However, analysis of raw data to end up in genome annotation or TSSs mapping is a demanding, time-consuming task, necessary for taking advantage of this information that may delay a more detailed analysis of specific issues. Among the large variety of these methods (see [70], [71] for review of most existing methods) a great amount of valuable information is obtained, resulting in highly efficient analysis of genome that, nonetheless, generally lacks a base on the physical mechanism of protein-DNA interaction. In this sense, our model and analysis method adopt a different strategy, not willing to compete in time performance with statistical-based techniques, but allowing a deeper understanding on the driving processes of protein binding. As a consequence of that, we are able not only to identify the TSSs, but also to characterize them in terms of physical magnitudes, allowing discussions about the strength of each site.

The nine promoters of cyanobacterium *Anabaena* PCC7120 studied in this work have been chosen in order to make the most of our model, without forgetting about its limitations. The genome of *Anabaena* PCC 7120 is well-known and the positions of TSSs have been defined under different metabolic conditions [72]. Firstly, it is remarkable how the different TSSs in the analyzed genes coincide with relevant states in the dynamics of the model, characterized as the heavier basins. In order to relate the information obtained with possible biological interpretation, we have analyzed a set of genes exhibiting several TSSs and whose regulation has been well characterized [67], [68], [73]–[77]. This choice allows us to assess directly the potential relation between the binding free energy values displayed in Table 1 for each of the located sites, and the relative strength of different TSSs associated to the same gene.

Among them, it is worth to mention the case of the *ntcA* promoter. The average opening shown in Fig. 3 reveals how the three existing TSSs in this 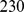 base pairs sequence [78] are clearly identified, agreeing also as sites which the particle visits with high probability. As displayed in Table 1, the relative free energy (with respect to the NS states) of the three TSSs is quite different. Indeed these values are in very good agreement with the occurrence and behavior of the three TSSs experimentally determined [78], [78]–[80]. TSS2, located at position 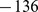, produces a constitutive transcript regardless of the culture conditions, while TSS1 (position 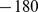) is only used in the absence of nitrogen. Finally TSS3 (position 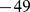) is also active under all conditions, but its use is highly induced under nitrogen deprivation. Table 1 displays a remarkably low free energy for TSS1, indicating that the presence of this macrostate is low in the dynamics, suggesting that its expression might be enhanced under more restrictive conditions. On the other hand, TSS2 and TSS3 appear as strong binding sites, covering both a large fraction of the total dynamics. These values are in good agreement with the *ntcA* transcription level at these sites under the correspondent conditions of nitrogen availability.

*FurB*, *petF* or *petH* show also consistent results. The TSSs of the three promotores are clearly identified, coinciding with the experimental positions [66], [72], [81]. Determination of TSSs for *FurB* promoter using the primer extension technique unravels revealing two TSSs at positions 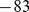 and 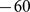 from the ATG, both with similar intensities ([66]). Our *in silico* analysis is in good concordance with such conclusions, as we find two major macrostates with very similar weight (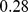 and 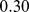) with an excitation just on these positions. The resulting profiles when the promoters of *petF* and *petH* are analyzed also display several preferred macrostates. Primer extension assays revealed a single TSS for the *petF* gene located at 100 bp upstream the translation start site [82]. More recently, high throughput analysis showed two TSSs for *petF*, at 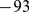 and 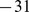, bp, in a better agreement with our predictions. Transcription of *petH*, encoding ferredoxin-NADP+ reductase takes place from a constitutive promoter at 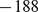 bp from the ATG and a NtcA activated promoter (TSS at 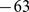 bp). According to the proposed model, both TSSs are found as relevant macrostates in the basin network, although not as high peaks in Fig. 3. Indeed, the constitutive TSS (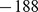) exhibits a higher probability (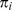) than the non-constitutive one (Table 1), indicating that the model is consistent with the experimental observations.

Concerning the five remaining promoters, high peaks are found around their single TSS, coinciding with the most (or one of the most) populated macrostates as we have defined them (Table 1). The case of *conR* is where our model works worse, as a significantly more relevant state appears in the dynamics. It should be noted that most experimentally determined TSSs have been obtained under standard culture conditions or under nitrogen deprivation, and the existence of additional TSSs under different conditions -impossible to account explicitly in our model- cannot be discarded. In addition, it must be noted that the model is not considering exclusively DNA-RNA polymerase interaction, but the influence of DNA breathing dynamics on protein binding. In such sense, additional binding sites for other proteins which are influenced by mechanical changes in the DNA conformation may also be detected.

We have compared our numerical results to the existing experimental ones on TSSs positions and intensities. Nonetheless, it is important to note that our method identifies additional relevant regions of the promoters that have not been experimentally probed yet. We shall mention the cases of promoters *furA*, *conR* or *nifB* where very populated macrostates appear aside from the discussed TSSs. Although we do not exclude the possibility of false positives, these macrostates may be related with unknown regulatory regions. Thus, our results suggest further experiments to search possible new relevant activity regions. Moreover, additional TSSs might appear if studied under different culture conditions, revealing the complexity of transcriptome profiles even in the case of simple organisms such as bacteria. To finish, we have already mentioned studies discussing the influence of bubble formation on certain DNA-binding proteins aside from RNA-polymerase [33], [34], [41], [42]. Being our model based on general physical features, additional macrostates found through our method might indicate the existence of binding sites for further regulatory proteins which participate in transcriptome processes of *Anabaena* PCC 7120.

*Anabaena* PCC 7120 has been shown to be an ideal experimental system to probe our numerical method. As it has been displayed, our results agree current experimental knowledge and propose possible new relevant activity regions. However, the model can be applied to the study of promoter sequences in many other organisms. Being the identification of protein binding sites in promoter sequences a key problem to understand and control regulation in biochemical and biotechnological processes, our methods appears as a powerful complementary tool in this scientific endeavor.

## Supporting Information

Text S1 This file contains the following information: (1) Explicit Langevin Equations for the model. (2) List of used parameters. (3) Further details on the analysis algorithm (construction of the CMN, SSD algorithm and free energy dendrograms construction. (4) Supplementary figures (dendrograms for the promoters not shown on the manuscript).
(PDF)
