# Supplementary material for: Mesoscopic Model and Free Energy Landscape for Protein-DNA Binding Sites: Analysis of Cyanobacterial Promoters
Source: PLoS Comput Biol. 2014 Oct 2;10(10):e1003835. doi: 10.1371/journal.pcbi.1003835 (PMC4183373; doi:10.1371/journal.pcbi.1003835)
Supplement: Text S1 — This file contains the following information: (1) Explicit Langevin Equations for the model. (2) List of used parameters. (3) Further details on the analysis algorithm (construction of the CMN, SSD algorithm and free energy dendrograms construction. (4) Supplementary figures (dendrograms for the promoters not shown on the manuscript). (PDF) [file pcbi.1003835.s001.pdf]

Supporting Information of "Mesoscopic model and free energy landscape for protein-DNA binding sites: analysis of Cyanobacterial promoters"

Rafael Tapia-Rojo, Juan José Mazo, Jose Ángel Hernández, María Luisa Peleato, Marí F. Fillat, and Fernando Falo

1 Materials and Methods (extended)

We extend the Materials and Methods section by writing explicitly the Langevin equations of motion and by explaining in a detailed way the analysis algorithm we use.

1.1 Langevin equations of motion

The model is simulated by integrating numerically the Langevin equation for both the chain base pairs and the particle. The explicit Langevin equations for the N base pairs are

$$m\frac{\partial^2 y_i}{\partial t^2} + m\eta\frac{\partial y_i}{\partial t} = -\frac{\partial\left[W(y_i, y_{i-1}) + W(y_i, y_{i+1})\right]}{\partial y_i} - \frac{\partial V}{\partial y_i} - \frac{\partial V_{int}}{\partial y_i} + \xi_i(t), \quad (1)$$

where η is the damping and $\xi_i(t)$ a white thermal noise (and so $\langle \xi_i(t)\rangle = 0$ and $\langle \xi_i(t)\xi_k(t')\rangle = 2m\eta k_B T\delta_{ik}\delta(t-t')$).

The particle follows

$$m_p\frac{\partial^2 X_p}{\partial t^2} + m_p\eta_p\frac{\partial X_p}{\partial t} = -\frac{\partial V_{int}}{\partial X_p} + \xi_p(t), \quad (2)$$

so analogously η_p stands for the damping and $\xi_p(t)$ for the thermal noise: $\langle \xi_p(t)\rangle = 0$ and $\langle \xi_p(t)\xi_p(t')\rangle = 2m_p\eta_p k_B T\delta(t-t')$.

1.2 Table of parameters

The parameters of the system are the following:

- **Base-pair parameters**: $m = 300\,Da$, $\eta = 5\,ps^{-1}$.
- **Intra base-pair potential**: $D_{AT} = 0.052\,eV$, $D_{CG} = 1.5D_{AT}$. $\alpha_{AT} = 4\,\mathring{A}^{-1}$, $\alpha_{CG} = 1.5\alpha_{AT}$. $G_x = 3D_x$, $y_{0x} = 2/\alpha_x$, $b_x = 0.5/\alpha_x^2$.
- **Inter base-pair potential**: $K = 0.03\,eV\mathring{A}^2$, $\rho = 3$, $\delta = 0.8\,\mathring{A}^{-1}$.
- **Particle parameters**: $M_p = 7000\,Da$, $\eta_p = 100\,ps^{-1}$.
- **Particle's potential parameters**: $B = 0.52\,eV$, $\gamma = 0.8\,\mathring{A}^{-1}$, $a = 1$, $\sigma = 3$.

1.3 Conformational Markov Network

The Conformational Markov Network (CMN) appears as a useful coarse-grained representation of large stochastic trajectories. This picture is obtained by discretizing the conformational space explored by the system and considering the dynamical jumps between the discretized configurations along the simulation. In this sense, the nodes of the complex network are defined by the discretized states, while the links account for the observed transitions between them. The arising network is thus a weighted and directed graph.

In our case, the conformational space is defined by the five first principal components, in order to reduce the number of degrees of freedom, keeping indeed the essential features of our system. We divide each of the principal component into 20 cells of equal volume, while the particle's trajectory is divided into N bins, coinciding with the N possible base pairs the particle can occupy. Our discretized conformational space is thus made up of $N \times 20^5$ possible states, which may be or not occupied within the stochastic trajectory. We assign each node a weight P_i accounting for the fraction of trajectory that the system has visited within the trajectory. The normalization condition $\sum_i P_i = 1$ holds. Secondly, the value P_{ij} is assigned to each directional link accounting for the dynamical jumps from node j to i. Self-loops can exist, and thus $P_{ii} \neq 0$. Finally the normalization condition $\sum_i P_{ij} = 1$ is forced. According to this, the CMN is totally defined by the occupancy vector $\mathbf{P} = \{P_i\}$ and the transition matrix $\tilde{S} = \{P_{ij}\}$. The matrix $\tilde{S}$ is the transition probability of the Markov chain defined by:

$$\mathbf{v}(t + \Delta t) = \tilde{S}\mathbf{v}(t), \tag{3}$$

where $\mathbf{v}(t)$ it the probability distribution at time t. If the trajectory is long enough, $\tilde{S}$ is ergodic and time invariant, vector $\mathbf{P}$ coincides with the stationary distribution associated with the Markov chain $\mathbf{P} = \tilde{S}\mathbf{P}$. Moreover, the detailed balance condition must hold:

$$P_{ji}P_i = P_{ij}P_j. \tag{4}$$

1.4 Stochastic Steepest Descent

Once we have translated de molecular dynamics trajectories onto a CMN, we apply the stochastic steepest descent (SSD) algorithm in order to split it into its basins of attraction in an efficient way, obtaining in turn useful thermo-statistical information about the system.The SSD algorithm is inspired in the deterministic steepest descent algorithm used to find minima in a multidimensional surface. We define the assisting vector $\mathbf{W} = \{w_i\}$, where i labels the nodes. The steps of the SSD algorithm are the following:

1. We start with $\mathbf{W} = \mathbf{0}$.
2. Select randomly a node l with $w_l = 0$ and write an auxiliary list of nodes adding l as first entry.
3. Select within the neighbors of l the node m that follows the maximum probability flux, this is $P_{ml} = \max\{P_{jl,\forall j \neq l}\}$. Check which of the following conditions is fulfilled:
   (a) If $P_{ml} > P_{lm}$ and $w_m = 0$, add m to the list and go back to 3. using m instead of l.
   (b) If $P_{ml} > P_{lm}$ and $w_m \neq 0$ write the labels of all the nodes in the list as $w_j = w_m$. Go back to step 3.
   (c) If $P_{ml} \leq P_{lm}$ remove link $l \to m$ from the graph. Return to point 3.

This process ends when every node in the CMN has been labelled, this is $w_i \neq 0 \forall i$. Then, the whole conformational space has been characterized and every node is connected with its local minima in the FEL. All nodes with the same label belong to the same basin in this FEL and therefore we can associate them with the same conformational state.

Given the basin partition, a new CMN network can be built, taken the basins themselves as new nodes. The occupation probabilities will now be defined as $P_\alpha = \sum_{i \in \alpha} P_i$, while the transition probabilities $P_{\beta\alpha} = \sum_{i \in \alpha} \sum_{j \in \beta} P_{ji} P_i / \sum_{i \in \alpha} P_i$. From this definitions rate constants can be easily calculated as $k_{\alpha\beta} = P_{\beta\alpha}/\Delta t$, while the relative free energy of basin α with respect to basin β is simply $\Delta F_\alpha = -k_B T \log(P_\alpha/P_\beta)$.

1.5 Free Energy dendrogram

The free energy dendrogram is a hierarchical representation of the FEL of the system that can be directly built from the basin structure. Taking $F/k_B T$ as previously defined as control parameter. We can reconstruct now the CMN by increasing gradually its value from an initial cut-off value. At each step, this cut-off is increased and new nodes emerge together with their links. This reconstruction provides a hierarchical picture of the nodes together with the way they are connected with each other. The graphical representation of this picture is the free energy dendrogram (also called disconnectivity graph), where each basin is represented in terms of its free energy and the barriers between basins represent the hierarchical relationship between each basin.

1.6 Definition of macrostates and non specific states

Considering the hierarchical free energy representation of the basin CMN we apply a new clustering procedure in order to define the macrostates of our system. This procedure is accomplished in order to provide a physical meaning to the states according to the purpose of our model. Typically, in our basin structure, we observe groups of basins which represent very similar physical states separated by small free energy barriers. It is plausible to think that such basins will be merged into a single state within short transition times.

The macrostates of the system are built according to this characteristic by clustering basins separated by barriers lower than $1.5 k_B T$. The system is able to jump thermally between these basins within short times, so we consider they represent the same physical state. The occupancies and transition probabilities of this clustered version of the basin CMN are constructed in an straight forward way.

The basin CMN structure shows another additional feature that allows us to distinguish between specific and non-specific states. Typically, up to the 90% of the network weight is distributed by less than the 1% of the basins. We name this large group of low populated basins as non-specific states. They represent short-lived transitionary states where the particle goes over the sequence searching for an stable binding site. The accumulated weight of these basins π_{NS} is considered to be that of the non-specific states and is used as reference value to calculate the free energy difference between an specific state i defined by its occupancy π_i and the non specific state $\Delta F_i / k_B T = \log \pi_i / \pi_{NS}$.

2 Supplementary figures

We include the free energy dendrograms for the six remaining promoters not shown in the main text.

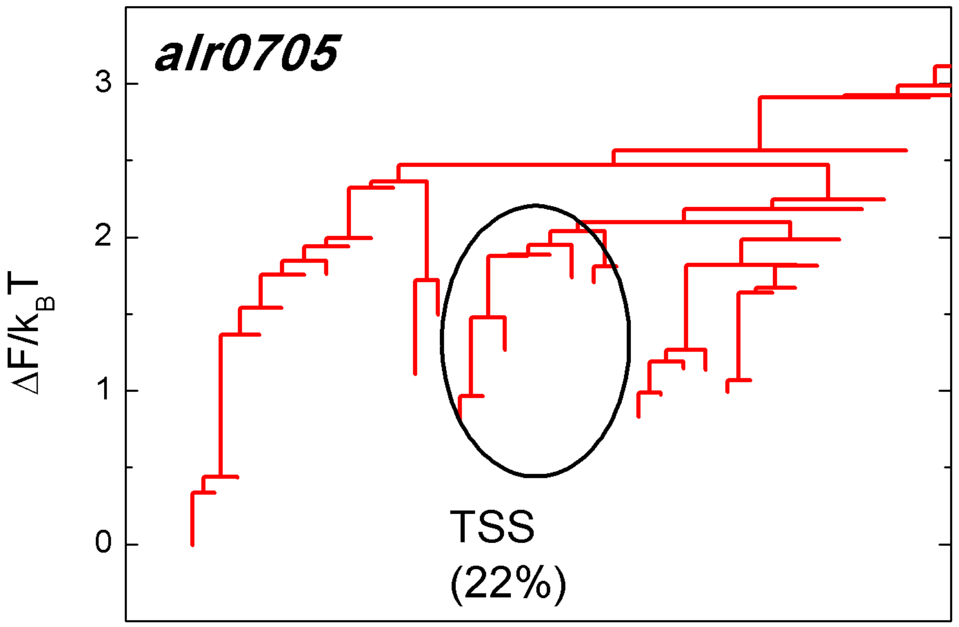

Figure 1: **Free energy dendrogram for *alr0705* promoter.** The states associated to the tss macrostates are rounded and their accumulated weight indicated.

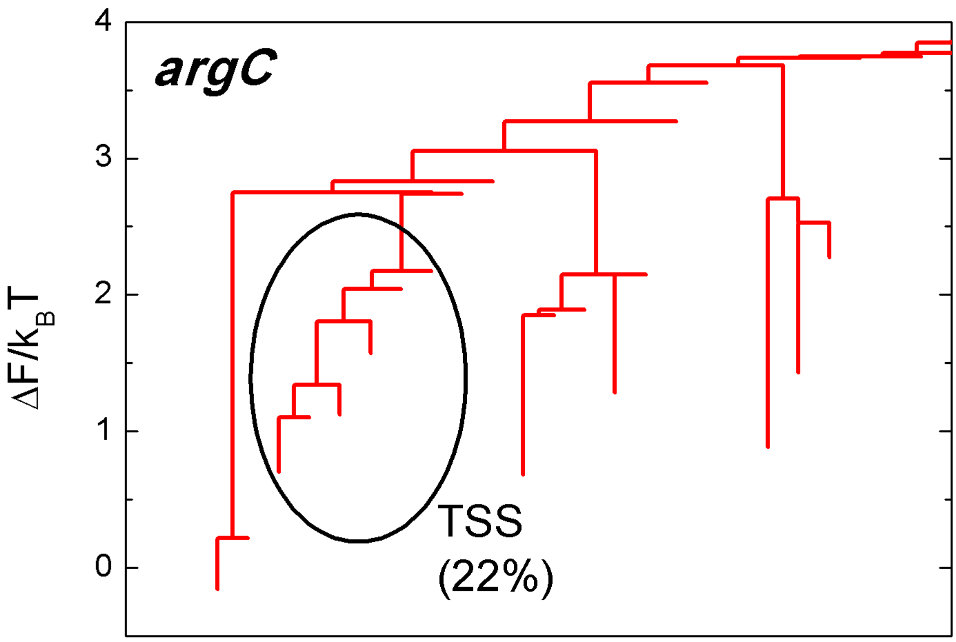

Figure 2: **Free energy dendrogram for *argC* promoter.** The states associated to the tss macrostate are rounded and their accumulated weight indicated.

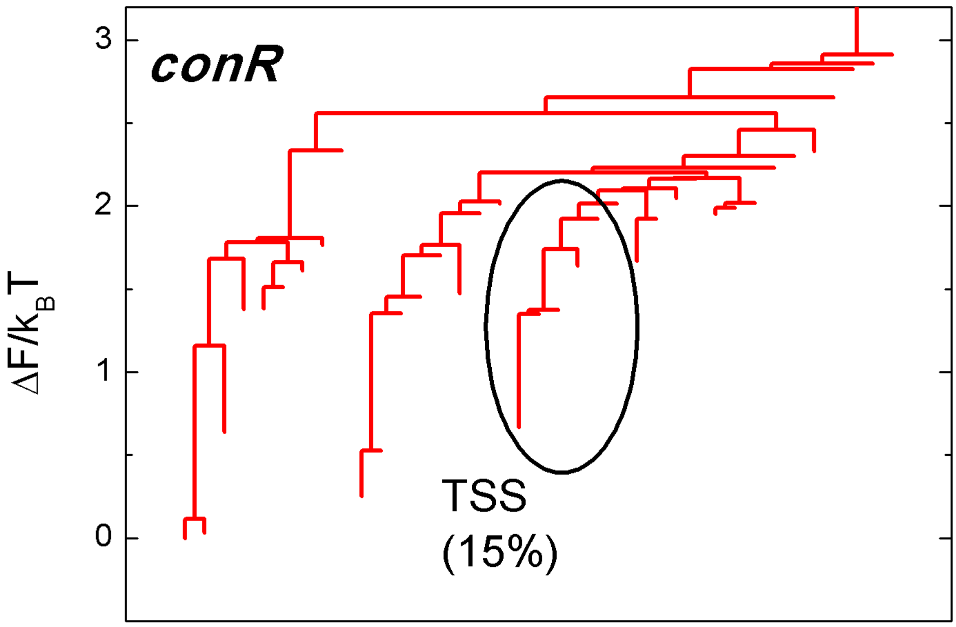

Figure 3: **Free energy dendrogram for *conR* promoter.** The states associated to the tss macrostate are rounded and their accumulated weight indicated.

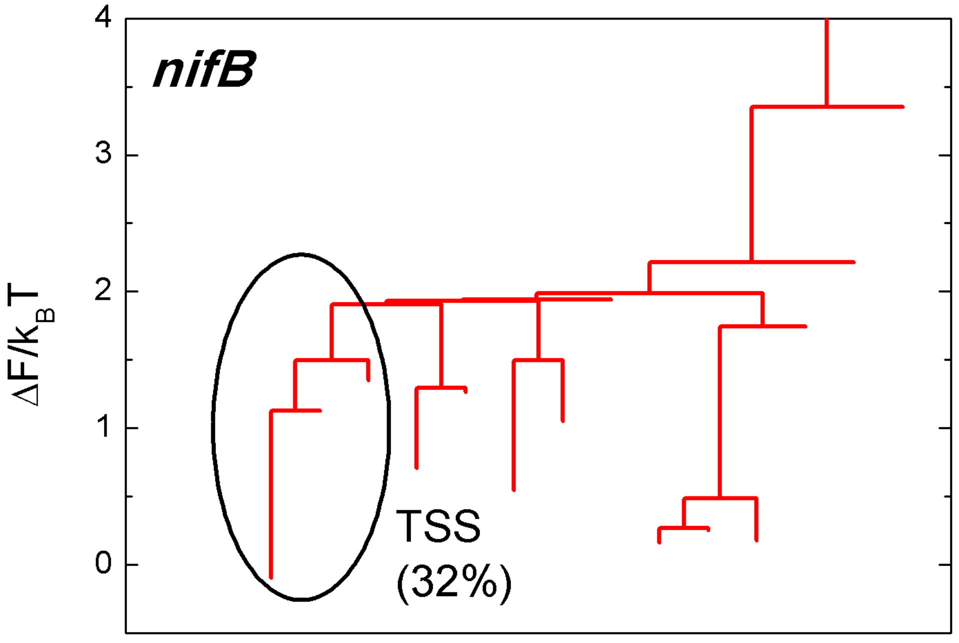

Figure 4: **Free energy dendrogram for *nifB* promoter.** The states associated to the tss macrostate are rounded and their accumulated weight indicated.

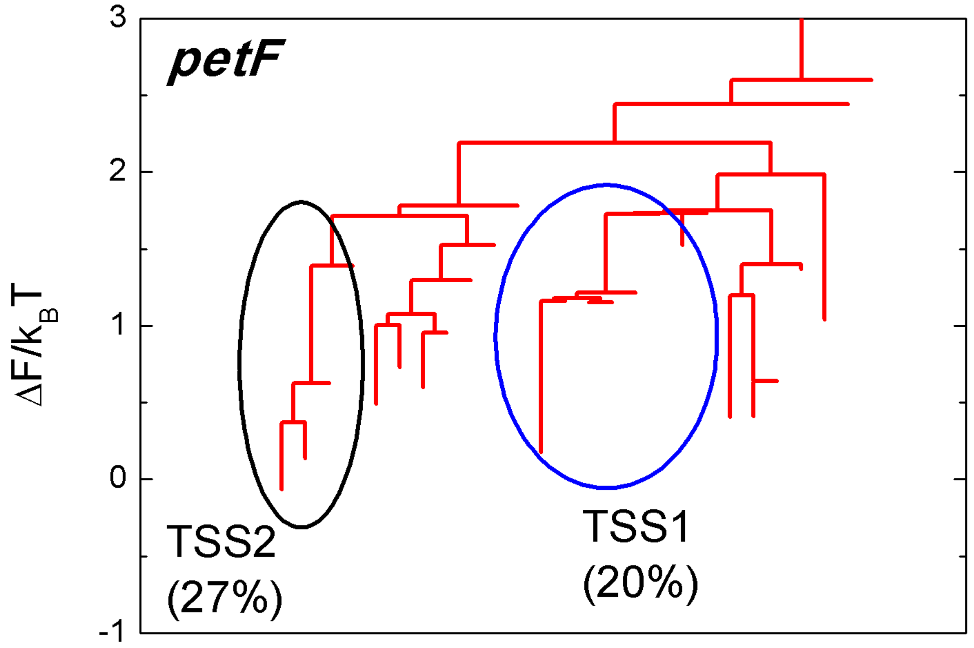

Figure 5: **Free energy dendrogram for *petF* promoter.** The states associated to the tss macrostates are rounded and their accumulated weight indicated.

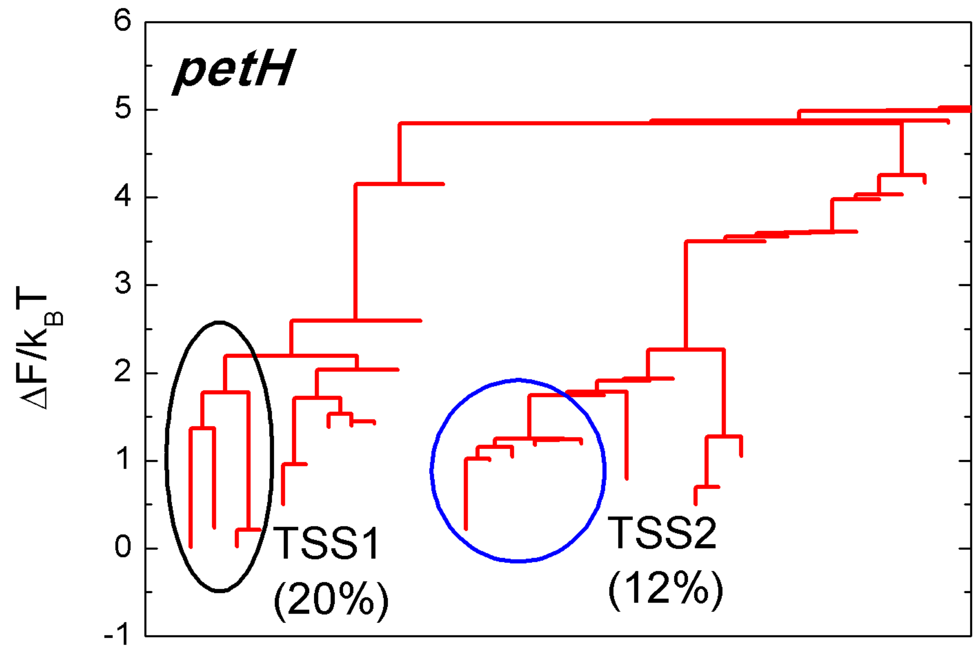

Figure 6: **Free energy dendrogram for *petH* promoter.** The states associated to the tss macrostates are rounded and their accumulated weight indicated.
